# Detection of Y Chromosome Microdeletions and Hormonal Profile Analysis of Infertile Men undergoing Assisted Reproductive Technologies

**DOI:** 10.22074/ijfs.2018.5244

**Published:** 2018-03-18

**Authors:** Ardeshir Bahmanimehr, Shahryar Zeighami, Bahia Namavar Jahromi, Zahra Anvar, Mohammad Ebrahim Parsanezhad, Maryam Davari, Somayeh Montazeri, Najmeh Moein Vaziri, Afsoon Zarei

**Affiliations:** 1 Thalassemia and Hemophilia Genetic, PND Research Center, Dastgheib Hospital, Shiraz University of Medical Science, Shiraz, Iran; 2 Infertility Research Center, Shiraz University of Medical Sciences, Shiraz, Iran; 3 Depatment of Urology, School of Medicine, Shiraz University of Medical Sciences, Shiraz, Iran; 4 Department of Obstetrics and Gynecology, School of Medicine, Shiraz University of Medical Sciences, Shiraz, Iran; 5 IVF Center, Ghadir Mother and Child Hospital, Shiraz University of Medical Sciences, Shiraz, Iran

**Keywords:** Assisted Reproductive Technologies, Non-obstructive Azoospermia, Y Chromosome Deletion

## Abstract

**Background:**

Y chromosome deletions (YCDs) in azoospermia factor (AZF) region are associated with ab- normal spermatogenesis and may lead to azoospermia or severe oligozoospermia. Assisted reproductive tech- nologies (ART) by intracytoplasmic sperm injection (ICSI) and testicular sperm extraction (TESE) are com- monly required for infertility management of patients carrying YCDs. The aim of this study was to estimate the frequency of YCDs, to find the most frequent variant in infertile men candidate for ART and to compare YCD distribution with a control fertile group. The semen parameters, hormonal profiles and ART outcomes of the infertile group were studied.

**Materials and Methods:**

This case-control study consisted of 97 oligozoospermic or non-obstructive azoospermic (NOA) infertile men, who had undergone ART, as the case group and 100 fertile men as the control group. DNA samples were extracted from blood samples taken from all 197 participants and YCDs were identified by multiplex polymerase chain reaction (PCR) of eight known sequence-tagged sites. The chi-square test was used to compare the mean values of hormone and sperm parameters between the two groups. P<0.05 was considered statistically significant.

**Results:**

No YCD was detected in the control group. However, 20 out of 97 (20.6%) infertile men had a YCD. AZFc, AZFbc and AZFabc deletions were detected in 15 (75%), four (20%) and one (5%) YCD-positive patients. No fer- tilization or clinical pregnancy was seen following ICSI in this sub-group with YCD. The mean level of FSH was significantly higher in the group with YCD (28.45 ± 22.2 vs. 4.8 ± 3.17 and 10.83 ± 7.23 in YCD-negative patients with and without clinical pregnancy respectively).

**Conclusion:**

YCD is frequent among NOA men and YCD screening before ART and patient counseling is thus strongly recommended.

## Introduction

Approximately 15% of all couples of reproductive age have difficulty conceiving a child (1). Male-factor infertility accounts for about half of these cases (2). Varicocele, obstruction of spermatic duct, erectile dysfunction, failure of ejaculation and sex hormone imbalances have been identified as major causes of male infertility. However, 10% of male infertility is due togenetic factors including chromosomal aneuploidiesand rearrangements, microdeletions and single gene defects (3, 4).

Proper spermatogenesis is dependent on numerousgenes, many of which are located on the long arm of theYchromosome (Yq11). A10 Mb region on the long armof the Ychromosome, namely the azoospermia factor(AZF) region, is frequently deleted in men with unex. plained spermatogenic failure. AZF was first mapped in1976 to Yq11 and has shown to play an important rolein male germ cell proliferation and differentiation (5,6). Further studies, by analyzing sequence-tagged sites (STS), have revealed the genetic complexity of the AZF region. This region is structurally subdivided into three sections, namely AZFa, AZFb and AZFc from proximal to distal of the Yq region (7). 

Yq has many palindrome repeats across the AZF. The homologous recombination between these repeats generates microdeletions in the AZF sub-regions, which in turn may lead to spermatogenic failure (8, 9). Y chromosome deletions (YCDs) may affect spermatogenesis at different progression steps. For example, deletion of AZFa causes Sertoli cell-only syndrome (SCOS). Identification of these deletions is thus highly important since isolating sperms by testicular sperm extraction (TESE) for intracytoplasmic sperm injection (ICSI) is improbable (10). Patients with AZFb microdeletions may have normal spermatogonia and primary spermatocytes in their tubules, however, they display pre-meiotic spermatogenic arrest or SCOS and, eventually, azoospermia. It is therefore difficult to recover mature sperms using TESE from AZFb-deleted patients (11, 12) . Complete deletion of AZFc, the most frequent type of YCD, presents a wide range of phenotypes from azoospermia to severe oligozoospermia (13, 14). 

The worldwide incidence of YCD is approximately 1-55.5% in infertile men showing significant variation among different populations (9). The aim of this study was to estimate the frequency of YCDs in infertile males and identify the most frequent variant among those who had ART at our Infertility Center and compared the results with those of a fertile group. Also semen parameters, hormonal profiles and ART outcomes of the infertile group were studied.

## Materials and Methods

### Patient selection and DNA extraction

In this case-control study, a total of 140 infertile men, from couples who had undergone ART at the Infertility Center of Ghadir Mother and Child Hospital, and 100 fertile men without any history of primary or secondary infertility and with at least one phenotypically normal child, who were referred to the Genetics Research Center at Shahid Dastghaib Hospital, were enrolled in this case-control study. 

The infertile men with known karyotype abnormalities, obstructive azoospermia, varicocele, testicular tumors and abnormal physical examinations were excluded, resulting in a case group consisting of 97 men. These individuals had either non-obstractive azoospermia (NOA) or oligozoospermia (defined as sperm counts less than 15×10^6^ according to the World Health Organization 2010. This study was approved by the Institutional Ethics Committee of Shiraz University of Medical Sciences and all of the participants signed a written consent form before enrolment. All 197 participants consciuosly donated a 2 ml peripheral blood sample and DNA was extracted from these samples by a commercial DNA extraction kit (Qiagen, Germany) and YCD typing was undertaken.

The hospital charts of the 97 infertile men were checked for their semen analysis parameters, hormonal profiles and ART outcomes. Semen samples had been analyzed for standard sperm quality parameters (volume, count, rates of motility and morphology) according to the World Health Organization (2010) and the Kruger classification (15, 16). ART outcomes were defined as fertilization and clinical pregnancy (CP). Fertilization was considered as development of two pro-nucleus stage embryos at 16-18 hours after in vitro fertilization (IVF)/ICSI. CP was defined as detection of a gestational sac and a fetal heart at 4-5 weeks after embryo transfer by transvaginal ultrasound scan.

### Polymerase chain reaction analysis

Samples were tested for classical YCD by typing six STSs, namely sY84, sY86, sY127, sY134, sY254 and sY255 by using the YChromStrip kit (Operon, Spain) for cases and a manual PCR method for all controls. Initially, the ZFX/ZFY was used to determine the presence of Y chromosome in all tested individuals. The detection of sY14 (SRY) was employed as an internal control of PCR. 

To detect AZFa, AZFb and AZFc, sY86, sY127 and sY254 were used for Multiplex PCR I and sY84, sY134 and sY255 were used for Multiplex PCR II. Multiplex PCR reactions were carried out in a total volume of 50 µL. Amplifications were carried out on a thermocycler (Eppendorf, Germany) with cycling conditions of an initial denaturation at 94°C for 15 minutes followed by 35 cycles of 94°C for 30 seconds for denaturation, 57°C for 90 seconds for primer annealing and 72°C for 1 minute for extension. This program was followed by a final extension step at 72°C for 10 minutes. A clear amplified product of the expected site was considered as a positive result for that site.

The reaction products were then analyzed by electrophoresis on a 1.5% agarose gel (Sigma, USA). If a deletion was observed, a second identical reaction was run to confirm the deletion in the presence of a positive control for that deletion. All primer sequences, the location of markers and the size of PCR products are shown (Table 1).

### Statistical analysis

The mean of follicle-stimulating hormone (FSH), luteinizing hormone (LH) and testosterone and sperm parameters were compared between groups using the t test (one-way ANOVA test considering number of the groups). P<0.05 was considered statistically significant.

**Table 1:** Details of primers, sequence-tagged sites (STS) location and polymerase chain reaction product sizes


Primer name	Sequence (5'-3')	Product size	Location	Acession number	Position

*ZFY*	F: GTCTTGTTGCAGCCCATGTA	495 bp	sY1301	BV679198.1	Yp11.2
	R: CAAAGGGAGAACTAGCAGGC				
*sY84*	F: AGAAGGGTCTGAAAGCAGGT	326 bp	DYS273	G12019	Yq11.1
	R: GCCTACTACCTGGAGGCTTC				
*sY86*	F: GTGACACACAGACTATGCTTC	320 bp	DYS148	G49207	Yq11.21
	R: ACACACAGAGGGACAACCCT				
*sY127*	F: GGCTCACAAACGAAAAGAAA	274 bp	DYS218	G11998	Yq11.222
	R: CTGCAGGCAGTAATAAGGGA				
*sY134*	F: GTCTGCCTCACCATAAAACG	301 bp	DYS224	G12001	Yq11.222
	R: ACCACTGCCAAAACTTTCAA				
*sY254*	F: GGGTGTTACCAGAAGGCAAA	380 bp	DAZ1	G38349	Yq11.223
	R: GAACCGTATCTACCAAAGCAGC				
*sY255*	F: GTTACAGCATTCGGCGTGAT	126 bp	DAZ	G65827	Yq11.223
	R: CTCGTCATGTGCAGCCAC				
*SRY*	F: GAATATTCCCGCTCTCCGGA	472 bp	SRY	G38356	Yp11.3
	R: GCTGGTGCTCCATTCTTGAG				


## Results

### Y chromosome deletion frequency

The 100 fertile men had a mean age of 29.67 ± 6.17 while the 97 infertile men with oligozoospermia or azoospermia had a mean age of 35.13 ± 7.7. No YCD was detected in the 100 fertile men. Twenty (20.6%) infertile men had YCD on their Yq. Of the observed YCD, AZFc was the most frequent (15 YCD-positive cases (75%), followed by AZFbc (four YCD-positive cases (20%) and AZFabc (singleton (5%).

### Assisted reproductive technologies outcome

We classified the infertile men who had ART based on presence/absence of YCD and clinical pregnancies into three groups. Number of participants and ART outcome results are shown (Table 2). 

**Table 2:** Classification of the infertile men based on presence/absence of YCD and clinical pregnancy after ART


Infertile men	Participants	Fertilization

CP in the absence of YCD	42	42
No CP in the absence of YCD	35	33
No CP in the presence of YCD	20	0


ART; Assisted reproductive technology, CP; Clinical pregnancy, and YCD; Y chromosome deletion. Values are presented as counts.

Sperm parameters, documented by semen analyses, of the three infertile groups and their hormonal profiles for the FSH, LH and testosterone are shown (Table 3). All patients carrying YCD were azoospermic. Moreover the mean level of FSH was significantly different between groups (P=0.023). The FSH level was 28.45 ± 22.2 in the group with YCD. However it was 4.8 ± 3.17 and 10.83 ± 7.23 in YCD-negative groups with or without clinical pregnancy respectively.

## Discussion

Microdeletions of the AZF region on the long arm of Y chromosome are one of the most important genetic causes of male infertility, which is manifested commonly as severe oligozoospermia and NOA (17, 18). The incidence of YCD is estimated to be about 65-70% in azoospermic men (19). Nevertheless, YCD has been reported to be approximately 5-13% in infertile men with severe oligozoospermia and azoospermia (20, 21).

There have been several studies reporting the prevalence of YCD in Iran, however, some discrepancies exist (22, 23). Totonchi et al. (24) investigated AZF microdeletions in 3654 Iranian infertile men. They found 185 cases (5.06%) with AZF microdeletions. Among patients carrying YCD, 79.4% had azoospermia and 20.5% had severe oligozoospermia. AZFc microdeletions were found to be the most prevalent form among YCDs. Recently, a meta-analysis conducted by Yousefi-Razin et al. (25) affirmed the rate of YCD to be 12.1% in Iranian azoospermic or severe oligozoospermic individuals. However, they suggested that the frequency of YCDs is related to ethnic and territorial differences.

**Table 3:** Sperm parameters and hormonal profiles of the infertile men in three groups


Infertile men	Volume (ml)	Count (×10^6^/ml)	Motility (%)	Morph (%)	FSH (mIU/ml)	LH (mIU/ml)	Testosterone (ng/dl)

CP and no YCD	3.72 ± 1.9	9.59 ± 1.8	23.64 ± 18.8	9.23 ± 6.1	4.8 ± 3.17	3.76 ± 2.5	4.53 ± 1.3
No CP and no YCD	3.29 ± 1.67	7.35 ± 1.8	20.91 ± 19.3	9.55 ± 5.4	10.83 ± 7.23	8.29 ± 7.8	6.63 ± 5.5
No CP and presence of YCD	4.17 ± 1.3	0	0	0	28.45 ± 22.2	8.56 ± 3.71	4.4 ± 2.6
P value	0.476	0.064	0.003^*^	0.45	0.023^*^	0.31	0.53


CP; Clinical pregnancy, YCD; Y chromosome deletion, Morph; Morphology, FSH; Follicle stimulating hormone, LH; Luteinizing hormone, and Testost; Testosterone. Values are presented as mean ± SD. *P<0.05 is significant.

In our study, YCD was detected in 20.6% of infertile men, among which the AZFc region deletions was the most frequent, comprising 75% of all deletions. These results are in agreement with previous reports in Iran and other countries (8, 14, 24, 26). We noticed that ICSI cycles undertaken for all of the 15 men carrying AZFc deletions failed in this study. This finding does not affirm the available literature which indicates that men with AZFc deletions may have successful ICSI outcomes (27). 

Previous studies have nevertheless shown that ICSI results are worse for men with NOA compared with men with obstructive azoospermia (20). Although the literature indicates that successful ART outcomes are possible after repeated ICSI cycles, the couple should be counseled about the inheritance of this fertility problem in their male offspring and sperm cryopreservation at a young age is strongly suggested for their male children.

It is reported that with complete deletion of AZFa and AZFb, TESE is usually not successful for sperm harvesting. Interestingly, one of our NOA patients who carried a complete AZFabc deletion had successful sperm retrieval by TESE. Although his retrieved sperm did not result in a successful fertilization, this finding shows that the results of YCD tests can not always predict the failure of sperm retrieval by TESE. We believe that ICSI failure in the YCD-positive sub-group in this study is likely due to their profound testicular failure which is reflected by their high mean FSH level (28.45 ± 22.2 mIU/ml).

All patients with YCD were azoospermic and had failed fertilization results after TESE and ICSI. There are several reports demonstrating a lower fertilization rate in patients with YCD compared with infertile men without any deletions (28-30). It should be taken into consideration that in the present study we did not select the patients according to presence/absence of YCD from the beginning. On the contrary, the infertile men who had ART were enrolled in this study. Although the study design here is different, the observation of no fertilization in the YCD-positive sub-group is in agreement with previous studies.

We observed a much higher FSH level in the group with YCDs compared with the other two groups. The appropriate induction and maintenance of sperm production is dependent on appropriate serum FSH levels. It has been shown that azoospermic men with FSH levels =20 IU/L have lower chances of having live-born children with the ICSI method (31, 32). YCDs cause impaired spermatogenesis and by inducing a positive feedback on FSH lead to higher FSH levels. Absence or severe reduction of spermatocytes has been known to cause high FSH levels. However, FSH levels are normal when there is normal sperm counts associated with maturation arrest. Till now, no cut-off value is identified for FSH that can accurately predict the failure of harvesting sperm during TESE.

Our second infertile group did not have any CP in spite of proceeding to the fertilization stage in 33 out of the 35 cases (94%) and being YCD-negative . Possibility of other concurrent causes of infertility such as poor oocyte quality in the two individuals who did not have fertilization should be considered. In the remaining 33 individuals, implantation issues may be a possible explanation for the failure of clinical pregnancy in fertilized oocytes.

Future studies regarding YCD frequencies and ART outcomes with larger sample sizes, in seprated ethnic groups are strongly recommended. Moreover, investigation of AZFc sub-mutations may lead to valuable insights. Also, evaluation of any probable correlation between YCD and histopathological results might add further insights. 

## Conclusion

YCD had a relatively high frequency among NOA men in our study. This result confirms the necessity of YCD screening for infertile men and appropriate patient counseling before ART.

## References

[1] de Kretser DM (1997). Male infertility. Lancet.

[2] Thonneau P, Marchand S, Tallec A, Ferial ML, Ducot B, Lansac J (1991). Incidence and main causes of infertility in a resident population (1,850,000) of three French regions (1988-1989). Hum Reprod.

[3] Vogt PH (2004). Molecular genetics of human male infertility: from genes to new therapeutic perspectives. Curr Pharm Des.

[4] Ambulkar PS, Sigh R, Reddy M, Varma PS, Gupta DO, Shende MR (2014). Genetic risk of azoospermia factor (AZF) microdeletions in idiopathic cases of azoospermia and oligozoospermia in central Indian population. J Clin Diagn Res.

[5] Tiepolo L, Zuffardi O (1976). Localization of factors controlling spermatogenesis in the nonfluorescent portion of the human Y chromosome long arm. Hum Genet.

[6] Vogt PH (2005). Azoospermia factor (AZF) in Yq11: towards a molecular understanding of its function for human male fertility and spermatogenesis. Reprod Biomed Online.

[7] Vogt PH, Edelmann A, Kirsch S, Henegariu O, Hirschmann P, Kiesewetter F (1996). Human Y chromosome azoospermia factors (AZF) mapped to different subregions in Yq11. Hum Mol Genet.

[8] Sheikhha MH, Zaimy MA, Soleimanian S, Kalantar SM, Rasti A, Golzade M (2013). Multiplex PCR screening of Y-chromosome microdeletions in azoospermic ICSI candidate men. Iran J Reprod Med.

[9] Suganthi R, Vijesh VV, Jayachandran S, Fathima Benazir JA (2013). Multiplex PCR based screening for microdeletions in azoospermia factor region of Y chromosome in azoospermic and severe oligozoospermic south Indian men. Iran J Reprod Med.

[10] Kleiman SE, Yogev L, Lehavi O, Hauser R, Botchan A, Paz G (2011). The likelihood of finding mature sperm cells in men with AZFb or AZFb-c deletions: six new cases and a review of the literature (1994-2010). Fertil Steril.

[11] Yu XW, Wei ZT, Jiang YT, Zhang SL (2015). Y chromosome azoospermia factor region microdeletions and transmission characteristics in azoospermic and severe oligozoospermic patients. Int J Clin Exp Med.

[12] Mau Kai C, Juul A, McElreavey K, Ottesen AM, Garn ID, Main KM (2008). Sons conceived by assisted reproduction techniques inherit deletions in the azoospermia factor (AZF) region of the Y chromosome and the DAZ gene copy number. Hum Reprod.

[13] Krausz C, Hoefsloot L, Simoni M (2014). Tuttelmann F, European Academy of Andrology, European Molecular Genetics Quality Network.EAA/EMQN best practice guidelines for molecular diagnosis of Y-chromosomal microdeletions: state-of-the-art 2013. Andrology.

[14] Elfateh F, Rulin D, Xin Y, Linlin L, Haibo Z, Liu RZ (2014). Prevalence and patterns of Y chromosome microdeletion in infertile men with azoospermia and oligzoospermia in Northeast China. Int J Reprod Med.

[15] Check JH, Adelson HG, Schubert BR, Bollendorf A (1992). Evaluation of sperm morphology using Kruger's strict criteria. Arch Androl.

[16] World Health Organization (2001). Laboratory manual of the WHO for the examination of human semen and sperm-cervical mucus interaction. Ann Ist Super Sanita.

[17] Zhu XB, Gong YH, He J, Guo AL, Zhi EL, Yao JE (2017). Multicentre study of Y chromosome microdeletions in 1,808 Chinese infertile males using multiplex and real-time polymerase chain reaction. Andrologia.

[18] Ferlin A, Arredi B, Speltra E, Cazzadore C, Selice R, Garolla A (2007). Molecular and clinical characterization of Y chromosome microdeletions in infertile men: a 10-year experience in Italy. J Clin Endocrinol Metab.

[19] Jungwirth A, Giwercman A, Tournaye H, Diemer T, Kopa Z, Dohle G (2015). European association of urology guidelines on male infertility: the 2012 update. Eur Urol.

[20] Reijo R, Alagappan RK, Patrizio P, Page DC (1996). Severe oligozoospermia resulting from deletions of azoospermia factor gene on y chromosome. Lancet.

[21] Malekasgar AM, Mombaini H (2008). Screening of Y chromosome microdeletions in iranian infertile males. J Hum Reprod Sci.

[22] Saliminejad K, Khorshid HR (2011). Discrepancy in the results of Y chromosome microdeletions in an Iranian population. J Hum Reprod Sci.

[23] Saliminejad K, Khorshid HR (2012). Methodological errors in screening of Yq microdeletion in Iranian azoospermic men. Indian J Med Res.

[24] Totonchi M, Mohseni Meybodi A, Borjian Boroujeni P, Sedighi Gilani M, Almadani N, Gourabi H (2012). Clinical data for 185 infertile Iranian men with Y-chromosome microdeletion. J Assist Reprod Genet.

[25] Yousefi-Razin E, Nasiri MJ, Omrani MD (2015). Frequency of Y chromosome microdeletions among Iranian infertile men with azoospermia and severe oligozoospermi: a meta-analysis. J Reprod Infertil.

[26] Zaimy MA, Kalantar SM, Sheikhha MH, Jahaninejad T, Pashaiefar H, Ghasemzadeh J (2013). The frequency of Yq microdeletion in azoospermic and oligospermic Iranian infertile men. Int J Reprod Med.

[27] Gardner D, Weissman A, Howles C, Shoham Z (2012). Textbook of assisted reproductive technologies.

[28] van Golde RJ, Wetzels AM, de Graaf R, Tuerlings JH, Braat DD, Kremer JA (2001). Decreased fertilization rate and embryo quality after ICSI in oligozoospermic men with microdeletions in the azoospermia factor c region of the Y chromosome. Hum Reprod.

[29] Choi JM, Chung P, Veeck L, Mielnik A, Palermo GD, Schlegel PN (2004). AZF microdeletions of the Y chromosome and in vitro fertilization outcome. Fertil Steril.

[30] Liu XH, Qiao J, Li R, Yan LY, Chen LX (2013). Y chromosome AZFc microdeletion may not affect the outcomes of ICSI for infertile males with fresh ejaculated sperm. J Assist Reprod Genet.

[31] Ramasamy R, Lin K, Gosden LV, Rosenwaks Z, Palermo GD, Schlegel PN (2009). High serum FSH levels in men with nonobstructive azoospermia does not affect success of microdissection testicular sperm extraction. Fertil Steril.

[32] Zitzmann M, Nordhoff V, von Schonfeld V, Nordsiek-Mengede A, Kliesch S, Schuring AN (2006). Elevated follicle-stimulating hormone levels and the chances for azoospermic men to become fathers after retrieval of elongated spermatids from cryopreserved testicular tissue. Fertil Steril.

